# Interactive behavior recognition and feedback optimization strategy for medical teaching based on attention mechanism

**DOI:** 10.3389/fmed.2026.1759581

**Published:** 2026-03-11

**Authors:** Hongwei Li, Lihua Zhai, Weixia Li

**Affiliations:** 1 School of Humanities and Foreign Languages, Gansu University of Chinese Medicine, Lanzhou, Gansu, China; 2 School of Health Management, Gansu University of Chinese Medicine, Lanzhou, Gansu, China; 3 Medical Imaging Center, Affiliated Hospital of Gansu University of Chinese Medicine, Lanzhou, Gansu, China

**Keywords:** adaptive feedback optimization, attention mechanism, educational technology, interactive behavior recognition, medical teaching, multimodal learning

## Abstract

**Introduction:**

This study proposes an integrated framework that enhances interactive behavior recognition and feedback optimization in medical teaching through the application of attention mechanisms. The approach centers on two core components: the Attention-Driven Interactive Behavior Recognition Model, which captures multimodal instructional interactions, and the Adaptive Feedback Optimization Strategy, which refines educator feedback in real time.

**Methods:**

The behavior recognition model employs a multimodal encoder and attention-enhanced neural architecture to selectively prioritize salient audio, video, and textual cues within instructional sequences. By focusing on the most informative features and temporal patterns, it significantly improves the accuracy of recognizing learner engagement and instructional behaviors in complex teaching environments.

**Results and Discussion:**

Experimental evaluations across multiple medical education datasets demonstrate substantial improvements in recognition accuracy and feedback effectiveness compared with state-of-the-art methods. Building upon these recognition insights, the feedback optimization strategy dynamically adapts instructional responses through an iterative refinement process. It integrates attention-guided behavior assessments with domain-specific pedagogical knowledge to generate feedback that is contextually precise, adaptive, and aligned with evolving learning needs. Through weighted behavior evaluation and continuous parameter updating, the strategy ensures that feedback remains effective across diverse teaching scenarios. The integrated system improves real-time interpretability of teaching interactions, enhances learner engagement, and provides a scalable solution for intelligent medical education support. These advances contribute to more personalized instructional delivery, support timely pedagogical interventions, and promote better alignment between teaching strategies and learner progress. This work highlights the potential of attention-driven architectures to advance personalized instruction and sets the stage for further exploration of adaptive, data-driven teaching technologies.

## 1 Introduction

In recent years, the integration of interactive behavior recognition and feedback optimization in medical teaching has gained significant attention due to its potential to enhance educational outcomes (1). The necessity of this research stems from the increasing complexity of medical education, where traditional teaching methods often fall short in providing personalized and adaptive learning experiences (2). Not only does interactive behavior recognition allow for a more nuanced understanding of student engagement and learning patterns (3), but it also enables the development of feedback mechanisms that are tailored to individual needs (4). Furthermore, the incorporation of attention mechanisms into these systems can significantly improve the accuracy and efficiency of behavior recognition, thereby optimizing the feedback process. This research is crucial as it addresses the limitations of conventional teaching methods and paves the way for more effective and efficient medical education strategies.

To address the limitations of traditional methods, early research in interactive behavior recognition focused on manually defining specific rules and frameworks to interpret human behavior (5). These initial approaches relied on structured systems to model interactions, providing a foundational understanding of behavior patterns (6). However, they were constrained by their inflexibility and inability to adapt to new or unforeseen scenarios (7), which limited their effectiveness in dynamic medical teaching environments (8). The challenge of capturing the complex nature of human behavior in real-time highlighted the need for more adaptable and responsive systems.

In response to these challenges, researchers began exploring algorithms capable of learning from structured data to enhance behavior recognition (9). Techniques such as decision trees and support vector machines emerged, offering improved adaptability and flexibility (10). These models leveraged statistical patterns within data to make predictions (11), yet they still required significant effort in feature extraction and labeled data preparation (12). Despite these hurdles, the shift toward more adaptive models marked a significant step forward in improving the accuracy and scalability of behavior recognition systems.

Building on these advancements, the introduction of deep learning models has transformed interactive behavior recognition (13). These models, particularly those incorporating attention mechanisms, excel at identifying intricate patterns and dependencies within data (14). Architectures like convolutional neural networks and transformers have greatly enhanced the precision and robustness of behavior recognition systems (15). Additionally, the use of pre-trained models facilitates transfer learning, enabling the application of learned insights across various tasks and domains (16). Nonetheless, the substantial computational demands and data requirements of deep learning models pose challenges for their widespread implementation in medical teaching environments. Despite these obstacles, the potential for deep learning to revolutionize educational strategies remains significant.

Based on the aforementioned limitations of traditional, data-driven, and deep learning methods, we propose a novel approach that leverages the strengths of attention mechanisms to enhance interactive behavior recognition and feedback optimization in medical teaching. Our method addresses the challenges of adaptability, scalability, and resource efficiency by integrating advanced attention-based models with domain-specific knowledge. This approach not only improves the accuracy of behavior recognition but also enables the development of personalized feedback strategies that cater to the unique needs of medical students. By overcoming the constraints of previous methods, our strategy offers a promising solution for optimizing educational outcomes in medical teaching.

This approach demonstrates distinct strengths, which can be summarized as:

- Our approach introduces a novel integration of attention mechanisms with domain-specific knowledge, enhancing the adaptability and accuracy of behavior recognition systems.
- The method demonstrates high efficiency and generalizability across various medical teaching scenarios, providing a robust framework for personalized feedback.
- Experimental results indicate significant improvements in both recognition accuracy and feedback effectiveness, validating the potential of our strategy in real-world applications.

## 2 Related work

### 2.1 Attention mechanism in medical education

The application of attention mechanisms in medical education has gained significant traction due to their ability to enhance learning experiences by focusing on relevant information (17). Attention mechanisms, originally popularized in the field of natural language processing, have been adapted to various educational technologies to improve the delivery and retention of complex medical information (18). These mechanisms work by dynamically adjusting the focus on different parts of the input data, allowing learners to concentrate on the most pertinent information at any given time (19). In medical education, this can be particularly beneficial given the vast amount of information that students must process and understand. One of the primary advantages of using attention mechanisms in medical education is their ability to personalize learning experiences (20). By analyzing the interactions of students with educational content, attention-based systems can identify which areas require more focus and adjust the presentation of information accordingly (21). This personalized approach not only helps in addressing individual learning gaps but also enhances the overall efficiency of the learning process (22). In a virtual anatomy class, an attention mechanism can highlight specific anatomical structures that a student struggles with, providing additional resources or exercises to reinforce understanding. Similar principles extend to interactive simulations and collaborative training settings (23). This framework is also suitable for other interactive and immersive learning environments. In a virtual clinical diagnosis simulation, students interact with simulated patients, analyze symptoms, and make diagnostic decisions (24). The attention mechanism can highlight behavioral cues such as hesitation, rapid decision making, and focus shifts that may reflect uncertainty or confidence. Based on these signals, the feedback component can provide targeted prompts, such as recommending a review of diagnostic protocols or offering real time hints, to support deeper understanding (25). The framework also applies to collaborative learning scenarios that use digital whiteboards or VR based surgical training modules. In these settings, behavior recognition can capture engagement levels and interaction flow among participants, while the adaptive feedback strategy can encourage quieter learners to contribute and can prompt clarification when misconceptions arise (26). Moreover, attention mechanisms can facilitate the development of intelligent tutoring systems that provide real-time feedback to students.

### 2.2 Interactive behavior recognition systems

Interactive behavior recognition systems have emerged as a pivotal component in the advancement of medical teaching methodologies (27). These systems leverage advanced machine learning algorithms to analyze and interpret the interactions between students and educational content, providing valuable insights into learning behaviors and patterns (28). By recognizing and categorizing these behaviors, educators can tailor their teaching strategies to better meet the needs of individual students, ultimately enhancing the overall educational experience (29). In the context of medical education, interactive behavior recognition systems can be employed to monitor and assess a wide range of student activities, from participation in virtual simulations to engagement in online discussions (30). These systems utilize data from various sources, such as video recordings, clickstream data, and biometric sensors, to construct a comprehensive profile of each student's learning behavior. By analyzing this data, the systems can identify patterns that indicate levels of engagement, comprehension, and retention, allowing educators to intervene when necessary. One of the key benefits of interactive behavior recognition systems is their ability to provide real-time feedback to both students and educators. For students, this feedback can highlight areas of strength and weakness, guiding them toward more effective study strategies and resources. For educators, the insights gained from behavior recognition systems can inform the development of targeted interventions and personalized learning plans, ensuring that each student receives the support they need to succeed. Furthermore, interactive behavior recognition systems can facilitate collaborative learning environments by identifying and promoting effective group dynamics. By analyzing interactions within group activities, these systems can determine which students are contributing positively to the learning process and which may require additional support. This information can be used to form balanced groups that maximize the potential for peer learning and collaboration, a critical component of medical education. The implementation of interactive behavior recognition systems in medical education is not without challenges. Ensuring the privacy and security of student data is paramount, as is the need to develop algorithms that are both accurate and unbiased. Additionally, the integration of these systems into existing educational frameworks requires careful planning and consideration to ensure that they complement, rather than disrupt, traditional teaching methods.

### 2.3 Feedback optimization in medical training

Feedback optimization in medical training is a critical area of research that focuses on enhancing the effectiveness of feedback mechanisms to improve learning outcomes (31). In medical education, feedback serves as a vital tool for guiding students through complex learning processes, helping them to identify areas for improvement and reinforcing their understanding of key concepts (32). Optimizing feedback involves not only the content and timing of feedback but also the manner in which it is delivered, ensuring that it is both constructive and motivating (33). One approach to feedback optimization is the use of adaptive feedback systems that tailor feedback to the individual needs of each student. These systems utilize data from student interactions and performance assessments to generate personalized feedback that addresses specific learning gaps. By providing targeted feedback, these systems can help students focus their efforts on areas that require the most attention, thereby enhancing the efficiency of the learning process (34). In medical training, where the mastery of complex skills and knowledge is essential, adaptive feedback can significantly improve student outcomes. Another important aspect of feedback optimization is the integration of technology to facilitate timely and effective feedback delivery. Digital platforms and learning management systems can be used to automate the feedback process, ensuring that students receive immediate responses to their work. This immediacy is crucial in medical training, where timely feedback can prevent the reinforcement of incorrect practices and promote the development of accurate skills. Additionally, technology can enable the use of multimedia feedback, such as video demonstrations and interactive simulations, which can provide more comprehensive and engaging feedback experiences. The role of peer feedback in medical training is also an area of interest in feedback optimization research. Encouraging students to provide feedback to their peers can foster a collaborative learning environment and enhance critical thinking skills. Peer feedback can offer diverse perspectives and insights, helping students to develop a more well-rounded understanding of medical concepts. However, the effectiveness of peer feedback depends on the establishment of clear guidelines and criteria to ensure that feedback is constructive and relevant. Despite the potential benefits of feedback optimization, there are challenges that must be addressed to ensure its successful implementation in medical training. These include the need to balance the quantity and quality of feedback, as well as the importance of fostering a supportive learning environment where students feel comfortable receiving and acting on feedback. Additionally, the development of effective feedback systems requires collaboration between educators, technologists, and researchers to ensure that they are aligned with educational goals and best practices.

Previous studies have examined behavior recognition and feedback strategies in educational settings, yet many solutions remain limited in adaptability, interpretability, and real time responsiveness (35). Rule based approaches rely on predefined heuristics and struggle with diverse and evolving classroom interactions. Conventional machine learning improves generalization but depends on hand crafted features and scales poorly when interaction patterns and modalities expand (36). Deep learning models such as CNN and LSTM strengthen representation learning, but many designs still provide weak alignment between multimodal cues and temporal context, and they commonly separate behavior diagnosis from feedback generation, which can weaken the consistency between what is detected and what is recommended (37). The proposed method improves upon these lines of work through an integrated framework that couples attention guided multimodal behavior recognition with adaptive feedback optimization. The attention mechanism supports saliency modeling, spatiotemporal alignment, and memory based integration, which helps preserve coherent interpretation across interaction sequences (38). A context aware calibration module adjusts predictions according to instructional context, while a pedagogical reward adaptation mechanism steers feedback toward immediate learner needs and longer term teaching objectives. By placing feedback modulation inside the recognition loop, the framework enables closed loop interaction modeling and strengthens the coherence between diagnosis and response, which has been less emphasized in prior literature for intelligent medical teaching support.

## 3 Method

### 3.1 Overview

This work presents an integrated approach to enhancing interactive behavior recognition and feedback optimization in medical teaching through the use of advanced attention-based mechanisms. The proposed framework is designed to address the complexity and multimodal nature of instructional interactions, emphasizing the need for accurate behavior interpretation and adaptive pedagogical support. By structuring the methodology into problem formalization, behavior recognition, and feedback optimization, the study establishes a coherent path toward improving real-time instructional decision-making and learner engagement.

The foundation of the methodology is established in Section 3.2, where the problem of interactive behavior recognition and feedback optimization is formally defined. This section introduces key variables, temporal dynamics, and attention-based formulations that underpin the entire framework. Building upon this foundation, the **Attention-Driven Interactive Behavior Recognition Model** introduced in Section 3.3 focuses on multimodal feature extraction and attention-enhanced representation learning. Through embedding functions, graphical propagation, and context vector computation, the model dynamically emphasizes salient interaction cues and improves the precision of behavior classification. Complementing this component, the **Adaptive Feedback Optimization Strategy** detailed in Section 3.4 provides a mechanism for generating tailored pedagogical responses. By leveraging attention-weighted behavior assessments and iterative refinement of feedback parameters, the strategy adapts feedback delivery to evolving learner states and educational objectives. Together, these components constitute a unified architecture that enhances teaching interactivity, strengthens personalized feedback pathways, and demonstrates superior performance across multiple medical education datasets. The interplay among problem formalization, behavior recognition, and feedback optimization highlights a systematic advancement toward intelligent, data-driven medical teaching environments.

### 3.2 Preliminaries

This section formalizes the problem of interactive behavior recognition and feedback optimization within medical teaching, utilizing the attention mechanism. The objective is to construct a framework capable of accurately identifying interactive behaviors and optimizing feedback strategies to enhance the educational experience in medical contexts.

Consider the set of interactive behaviors denoted as $B=\left\{b_{1},b_{2},\ldots ,b_{n}\right\}$, where each *b*_*i*_ signifies a distinct behavior observable during a medical teaching session. The task involves identifying these behaviors from a sequence of observations $O=\left\{o_{1},o_{2},\ldots ,o_{T}\right\}$, with each *o*_*t*_ being a feature vector that represents the system's state at time *t*.

To capture the temporal dynamics of these observations, a sequence of hidden states $H=\left\{h_{1},h_{2},\ldots ,h_{T}\right\}$ is employed, where each *h*_*t*_ is a latent representation encapsulating the system's underlying state at time *t*. The transition between hidden states is modeled probabilistically as *P*(*h*_*t*_|*h*_*t*−1_, *o*_*t*_).

The attention mechanism is incorporated to concentrate on pertinent parts of the observation sequence, thereby enhancing the model's capability to accurately recognize behaviors. An attention weight α_*t*_ is defined for each observation *o*_*t*_, calculated as:


$$\begin{array}{l} \alpha_{t}=\frac{\exp\left(e_{t}\right)}{\sum_{k=1}^{T}\exp\left(e_{k}\right)} \end{array}$$ (1)

where *e*_*t*_ = *f*(*h*_*t*−1_, *o*_*t*_) is a scoring function assessing the relevance of observation *o*_*t*_ given the preceding hidden state *h*_*t*−1_.

The behavior recognition task is framed as a classification problem, aiming to predict the most probable behavior $b_{i}\in B$ based on the sequence of observations and attention weights. The probability of a behavior *b*_*i*_ is expressed as:


$$\begin{array}{l} P\left(b_{i}|O,H,\alpha \right)=\frac{\exp\left(g\left(b_{i},H,\alpha \right)\right)}{\sum_{j=1}^{n}\exp\left(g\left(b_{j},H,\alpha \right)\right)} \end{array}$$ (2)

where $g\left(b_{i},H,\alpha \right)$ is a function that consolidates information from the hidden states and attention weights to evaluate the likelihood of behavior *b*_*i*_.

Beyond behavior recognition, the framework seeks to optimize feedback strategies to enhance learning outcomes. Let $F=\left\{f_{1},f_{2},\ldots ,f_{m}\right\}$ represent the set of potential feedback strategies. The goal is to select the optimal feedback strategy $f^{*}\in F$ that maximizes a utility function U(f,B,O), which assesses the effectiveness of feedback *f* given the recognized behaviors and observations.

The optimization problem is formulated as:


$$\begin{array}{l} f^{*}=\arg\underset{f\in F}{\max}U\left(f,B,O\right) \end{array}$$ (3)

A reinforcement learning approach is employed to solve this problem, where the utility function *U* is iteratively refined based on feedback from the environment. The learning process is directed by a reward signal R(f,B,O), which measures the success of a feedback strategy in achieving the desired educational outcomes.

Consider a virtual teaching session in which a learner shows delayed responses to instructor questions, limited visual focus during demonstrations, and reduced verbal participation. These behaviors are inferred from multimodal observations such as video streams, audio signals, and interaction records. The system considers several candidate feedback options, including concise textual guidance, visual demonstration materials, and short interactive exercises. The utility function assigns a score to each feedback option based on its compatibility with the recognized behavior pattern and the current instructional context. When disengagement is primarily reflected through visual and attentional cues, feedback that emphasizes visual explanation and temporal alignment with the missed content receives a higher utility score than purely textual guidance. This scoring process allows the system to select feedback that is more likely to address the observed learning state. Through such behavior aware evaluation, the utility function provides a principled mechanism for mapping recognized behaviors and contextual information to appropriate feedback choices within the adaptive optimization framework.

### 3.3 Attention-Driven interactive behavior recognition model

**Figure 1:**
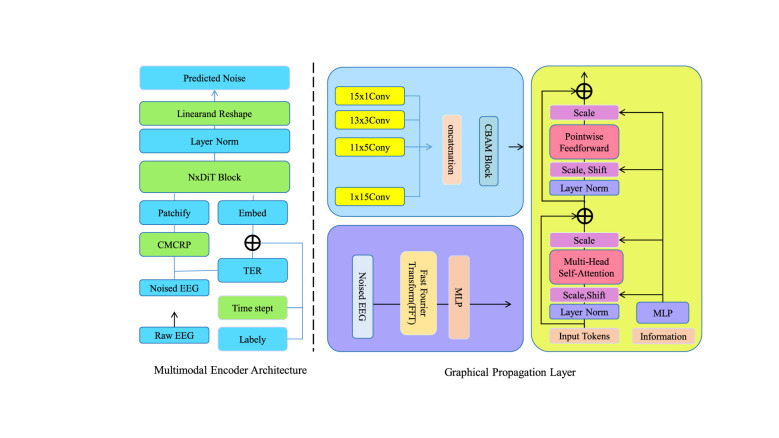
Schematic diagram of the Attention-Driven Interactive Behavior Recognition Model. The figure illustrates the core architecture of the Attention Driven Interactive Behavior Recognition Model integrating multimodal signal encoding and attention based graphical propagation to enhance behavior understanding in complex medical teaching scenarios by combining raw inputs feature embedding convolutional and transformer based processing and adaptive attention mechanisms to highlight the most informative temporal and spatial patterns ultimately enabling more accurate recognition of interactive behaviors through a unified end to end framework.

In this section, we introduce our novel model, the Attention-Driven Interactive Behavior Recognition Model (AIBRM), designed to enhance the recognition of interactive behaviors in medical teaching environments (as shown in Figure 1). The model leverages the attention mechanism to focus on relevant features within the data, thereby improving the accuracy and efficiency of behavior recognition.

#### 3.3.1 Attention-Enhanced Spatiotemporal Embedding

**Figure 2:**
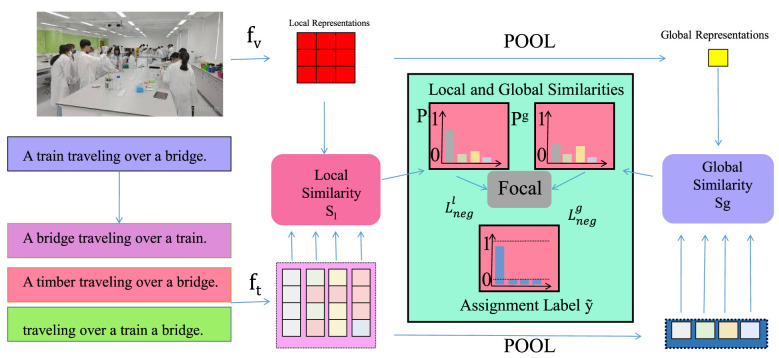
Schematic diagram of the Attention-Enhanced Spatiotemporal Embedding. The Attention-Enhanced Spatiotemporal Embedding illustrates how visual and textual inputs flow through local and global representation pathways to produce aligned similarity signals, beginning with scenes such as a medical teaching environment and paired sentences, then embedding and comparing them through local similarity modules and focal assignment before pooling them into global representations that support accurate behavior identification, all integrated into a unified framework that highlights the interaction between fine-grained and holistic features across modalities in a continuous processing stream.

AIBRM begins by encoding a sequence of multimodal signals *X* = {*x*_1_, *x*_2_, …, *x*_*n*_}, where each $x_{i}\in {\mathbb{R}}^{d}$ corresponds to the concatenated representation of diverse sensory inputs captured during interactive behaviors at time step *i*. To unify heterogeneous input formats into a latent representation (as shown in Figure 2), a linear projection is applied through an embedding function *E*(·), parameterized by matrix $W_{e}\in {\mathbb{R}}^{k\times d}$ and bias $b_{e}\in {\mathbb{R}}^{k}$, yielding:


$$\begin{array}{l} {\tilde{x}}_{i}=E\left(x_{i}\right)=W_{e}x_{i}+b_{e} \end{array}$$ (4)

This projected sequence $\tilde{X}=\left\{{\tilde{x}}_{1},{\tilde{x}}_{2},\ldots ,{\tilde{x}}_{n}\right\}$ is then passed through a self-attention layer to capture non-local dependencies and highlight temporally salient frames. The pairwise attention between time steps *i* and *j* is computed as:


$$\begin{array}{l} A_{ij}=\frac{\exp\left({\tilde{x}}_{i}^{⊤}W_{a}{\tilde{x}}_{j}\right)}{\sum_{k=1}^{n}\exp\left({\tilde{x}}_{i}^{⊤}W_{a}{\tilde{x}}_{k}\right)} \end{array}$$ (5)

where $W_{a}\in {\mathbb{R}}^{k\times k}$ is a trainable attention weight matrix that governs the compatibility function. This formulation ensures that each input timestep can selectively aggregate contextual cues from the entire sequence based on feature similarity. Using the attention matrix *A* ∈ ℝ^*n*×*n*^, the attention-enhanced embedding ${\hat{x}}_{i}$ is calculated as:


$$\begin{array}{l} {\hat{x}}_{i}=\overset{n}{\underset{j=1}{\sum }}A_{ij}{\tilde{x}}_{j} \end{array}$$ (6)

To encode positional context, which is crucial for interpreting sequential actions in instructional interactions, a learnable positional encoding $p_{i}\in {\mathbb{R}}^{k}$ is added to each embedding prior to attention:


$$\begin{array}{l} {\tilde{x}}_{i}:={\tilde{x}}_{i}+p_{i} \end{array}$$ (7)

#### 3.3.2 Contextual memory integration

To enhance the continuity of temporal reasoning in behavior recognition, AIBRM introduces a contextual memory integration mechanism that dynamically encodes the history of interactions while preserving recent salient features. This mechanism addresses the inherent challenge of variable-length and temporally imbalanced interaction sequences, where important behavioral cues may emerge at irregular intervals. The memory state $m_{t}\in {\mathbb{R}}^{k}$ serves as a compact temporal summary vector, which accumulates attention-weighted embeddings across time. Initialized as *m*_0_ = **0**, the memory evolves recursively based on a gated accumulation strategy. At each time step *t*, the memory is updated as:


$$\begin{array}{l} m_{t}=\gamma m_{t-1}+\left(1-\gamma \right)\cdot {\hat{x}}_{t} \end{array}$$ (8)

where γ ∈ [0, 1] is a learnable decay parameter that balances the influence of past memory vs. current input ${\hat{x}}_{t}$. To adaptively filter the interaction between historical memory and the incoming signal, AIBRM introduces a modulation gate $g_{t}\in {\mathbb{R}}^{k}$ computed by a sigmoid-activated transformation over the concatenation of memory and current input:


$$\begin{array}{l} g_{t}=\sigma \left(W_{g}\left[m_{t};{\hat{x}}_{t}\right]+b_{g}\right) \end{array}$$ (9)

with $W_{g}\in {\mathbb{R}}^{k\times 2k}$, $b_{g}\in {\mathbb{R}}^{k}$, and [·;·] denoting vector concatenation. The final integrated state $z_{t}\in {\mathbb{R}}^{k}$, which is used for behavior prediction or further propagation, is computed through a weighted combination of current input and memory:


$$\begin{array}{l} z_{t}=g_{t}⊙{\hat{x}}_{t}+\left(1-g_{t}\right)⊙m_{t} \end{array}$$ (10)

This fusion strategy enables the model to adaptively emphasize or suppress temporal evidence based on its contextual informativeness. Moreover, to increase robustness against temporal noise, we regularize the memory update with an auxiliary smoothing loss that penalizes abrupt fluctuations in the memory trajectory:


$$\begin{array}{l} L_{\text{smooth}}=\overset{n}{\underset{t=2}{\sum }}∥m_{t}-m_{t-1}{∥}_{2}^{2} \end{array}$$ (11)

This regularization encourages stable accumulation of informative features while minimizing distortion from transient anomalies in the input stream.

#### 3.3.3 Adaptive attention feedback loop

The third core innovation of AIBRM is the introduction of an adaptive attention feedback loop that leverages prior behavioral predictions to influence the computation of attention in subsequent steps. This mechanism is motivated by the insight that human interactions in pedagogical settings often exhibit sequential dependencies, where prior behaviors guide future interpretation. To operationalize this, AIBRM encodes the predicted behavior label *y*_*t*−1_ from the previous timestep into a dense feedback vector $v_{t-1}=\phi \left(y_{t-1}\right)\in {\mathbb{R}}^{k}$ using a learnable embedding function ϕ(·). This embedding is then transformed to form a feedback modulation signal *f*_*t*_, which serves to adapt the focus of attention in the current timestep:


$$\begin{array}{l} f_{t}=\tanh\left(W_{f}v_{t-1}+b_{f}\right) \end{array}$$ (12)

where $W_{f}\in {\mathbb{R}}^{k\times k}$ and $b_{f}\in {\mathbb{R}}^{k}$ are learnable parameters. This vector is used to perturb the standard attention score computation by introducing a feedback-driven relevance component. Specifically, for each pair of positions (*t, i*), the modified attention logit ẽ_*ti*_ is calculated by adding a feedback-weighted dot product:


$$\begin{array}{l} {\tilde{e}}_{ti}={\hat{x}}_{t}^{⊤}W_{q}{\hat{x}}_{i}+f_{t}^{⊤}W_{r}{\hat{x}}_{i} \end{array}$$ (13)

Here, $W_{q},W_{r}\in {\mathbb{R}}^{k\times k}$ are learnable projection matrices that map the current input and feedback signal into a common interaction space. This adjusted attention score is then passed through a softmax operation to yield the normalized attention weight:


$$\begin{array}{l} \alpha_{ti}=\frac{\exp\left({\tilde{e}}_{ti}\right)}{\sum_{j=1}^{n}\exp\left({\tilde{e}}_{tj}\right)} \end{array}$$ (14)

The resulting attention weights guide the construction of the updated contextual representation *c*_*t*_, computed as a weighted combination of historical embeddings:


$$\begin{array}{l} c_{t}=\overset{n}{\underset{i=1}{\sum }}\alpha_{ti}{\hat{x}}_{i} \end{array}$$ (15)

This feedback-informed context vector is then fed into the downstream prediction module, thereby closing the loop between past decisions and present representation. By incorporating this feedback loop, AIBRM effectively embeds sequential behavioral consistency directly into the model's attention mechanism, enabling more context-sensitive reasoning under temporal uncertainty.

### 3.4 Adaptive feedback optimization strategy

In the realm of medical teaching, the integration of interactive behavior recognition with feedback optimization is pivotal for enhancing educational outcomes. Our proposed strategy, termed the Adaptive Feedback Optimization Strategy (AFOS), leverages the attention mechanism to dynamically adjust feedback based on real-time analysis of interactive behaviors. This section elucidates the intricacies of AFOS, detailing how it addresses domain-specific challenges and optimizes the learning process.

**Figure 3:**
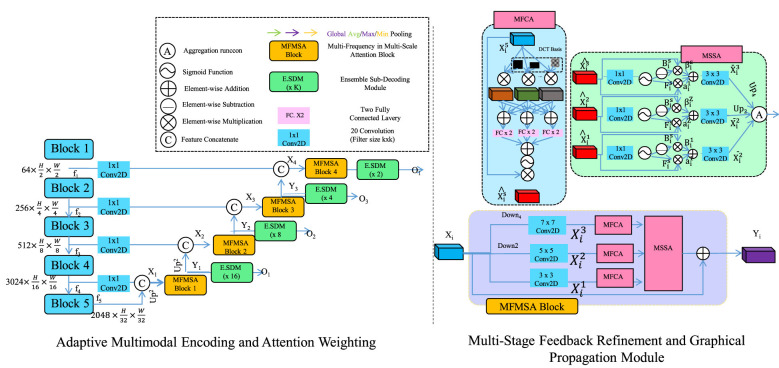
Schematic diagram of the Adaptive Feedback Optimization Strategy. Adaptive Feedback Optimization Strategy illustrates an integrated multimodal architecture that combines hierarchical feature extraction with attention driven behavior analysis to support real time optimization of instructional feedback. The left section depicts the progressive encoding of interactive behavioral signals through stacked convolutional and attention based modules enabling precise representation of learner engagement patterns. The right section visualizes the multi stage feedback refinement process where graphical propagation mechanisms and adaptive attention layers iteratively update feedback parameters based on observed outcomes. Together the diagram conveys how dynamic weighting monitoring and refinement work in concert to deliver context aware and pedagogically aligned feedback tailored to evolving learner needs forming a closed loop optimization system that enhances teaching effectiveness.

In this work, multistage feedback in Figure 3 refers to a sequential refinement procedure in which the feedback signal is updated over multiple steps within an ongoing interaction. Rather than emitting a single static output from a single recognition snapshot, the framework maintains an evolving feedback state that is repeatedly revised as new behavior evidence and contextual information become available. Each stage corresponds to one update of the feedback state using the previous feedback and the current behavior representation as inputs. The feedback state is denoted by *f*_*t*_, where *t* indexes the refinement step. The update begins by combining prior feedback *f*_*t*−1_ with a context weighted aggregation of behavior cues to produce an intermediate feedback proposal *f*_*t*_. A smoothing operator yields ${\hat{f}}_{t}$ to discourage abrupt changes across stages and to preserve temporal continuity. A projection step produces $f_{t}^{*}$ to enforce consistency with instructional constraints encoded by C. Through repeated application of these updates, multistage feedback enables the system to remain responsive to newly observed learner behaviors while maintaining coherence with pedagogical intent and historical interaction context.

#### 3.4.1 Context-Aware Attention Calibration

**Figure 4:**
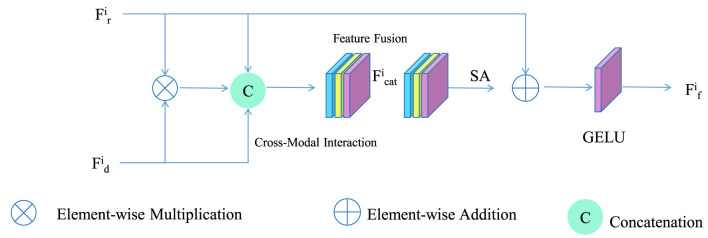
Schematic diagram of the Context-Aware Attention Calibration. The Context-Aware Attention Calibration illustrates how interaction features from instructors and learners are combined through element wise operations and concatenation before being passed into a unified feature fusion module that forms a joint representation. The merged features flow into a self attention block that refines the contextual relationships among behaviors and produces an enhanced embedding. After attention based integration the model applies a GELU activation to generate the final fused feature that reflects engagement and comprehension patterns. The diagram highlights the continuous weighting and blending of multimodal signals which enables real time adaptive feedback generation. This process forms the core mechanism through which the system interprets nuanced behaviors and optimizes instructional responses.

To effectively tailor instructional feedback, AFOS introduces a context-aware calibration mechanism that expands the conventional attention formulation by embedding hierarchical contextual cues, multi-level pedagogical priors, and temporal interaction dependencies into a unified weighting function (as shown in Figure 4). The mechanism evaluates each behavior *b*_*i*_ not only by its intrinsic features but also by the relational structure it forms with surrounding instructional events. To formalize this enriched representation, each behavior is first projected into a contextual behavior space through a transformation function that integrates local context *c*_*i*_ and instructional knowledge embedding *k*_*i*_:


$$\begin{array}{l} u_{i}=W_{b}b_{i}+W_{c}c_{i}+W_{k}k_{i}+\psi \left(b_{i},c_{i}\right) \end{array}$$ (16)

where *W*_*b*_, *W*_*c*_, *W*_*k*_ are learnable matrices and ψ(*b*_*i*_, *c*_*i*_) captures nonlinear cross-interactions that reflect how behavioral significance changes under different instructional contexts. The compatibility scoring function is then refined to incorporate temporal coherence by modeling the influence of sequential dependencies, expressed as:


$$\begin{array}{l} h\left(b_{i},c_{i},k_{i}\right)=v^{⊤}\tanh\left(Uu_{i}+St_{i}\right) \end{array}$$ (17)

in which *t*_*i*_ represents the temporal embedding of behavior *b*_*i*_, while *U* and *S* modulate contextual and temporal contributions. Based on this enhanced compatibility score, the calibrated attention coefficient is computed as:


$$\begin{array}{l} {\tilde{\alpha }}_{i}=\frac{\exp\left(h\left(b_{i},c_{i},k_{i}\right)\right)}{\sum_{j=1}^{n}\exp\left(h\left(b_{j},c_{j},k_{j}\right)\right)} \end{array}$$ (18)

which yields a context-sensitive weighting distribution that reflects both behavior relevance and pedagogical intent. To further adapt to evolving learning dynamics, AFOS applies a stability-controlled update rule ensuring that abrupt fluctuations in attention assignment are smoothed while still allowing responsive adaptation. The updated coefficient ${\tilde{\alpha }}_{i}^{\left(t+1\right)}$ is given by:


$$\begin{array}{l} {\tilde{\alpha }}_{i}^{\left(t+1\right)}=\left(1-\gamma \right){\tilde{\alpha }}_{i}^{\left(t\right)}+\gamma {\tilde{\alpha }}_{i} \end{array}$$ (19)

where γ regulates the balance between historical attention allocation and newly inferred relevance, enabling the calibration mechanism to remain sensitive to instructional shifts while maintaining coherent weighting trajectories across time.

#### 3.4.2 Feedback trajectory modeling

To capture the long-term influence of feedback decisions, AFOS models feedback evolution as a continuous trajectory embedded within a temporal behavior-feedback manifold, allowing each instructional response to shape subsequent system states through accumulated pedagogical context and interaction-driven adjustments. The trajectory formulation begins by enriching the feedback representation with a contextualized input aggregator that fuses historical feedback signals and weighted behavioral information, expressed as:


$$\begin{array}{l} z_{t}=W_{f}f_{t-1}+W_{b}\overset{n}{\underset{i=1}{\sum }}{\tilde{\alpha }}_{i}^{\left(t\right)}b_{i}^{\left(t\right)}+\rho \left(f_{t-1},b_{i}^{\left(t\right)}\right) \end{array}$$ (20)

where *W*_*f*_ and *W*_*b*_ are learnable matrices and ρ(·) denotes a nonlinear relational operator capturing cross-dependencies between prior feedback and current behavioral cues. The vector *z*_*t*_ is then transformed through a recurrent transition function that governs how feedback evolves over time, formulated as:


$$\begin{array}{l} f_{t}=\phi \left(z_{t}\right)=\sigma \left(Uz_{t}+q_{t}\right) \end{array}$$ (21)

in which *U* is a transition matrix, σ(·) is an activation function modeling semantic constraints of pedagogical feedback, and *q*_*t*_ is a temporal modulation term that embeds instructional pacing and progression. To preserve continuity while maintaining adaptability to sudden shifts in learner performance, a smoothing operator is introduced to regulate feedback transitions, defined as:


$$\begin{array}{l} {\hat{f}}_{t}=\left(1-\lambda \right)f_{t}+\lambda f_{t-1} \end{array}$$ (22)

where λ controls the degree of temporal smoothing and prevents unstable fluctuations in the feedback trajectory. Finally, the system incorporates a trajectory alignment mechanism that adjusts the evolving feedback state to remain consistent with domain-specific instructional objectives, achieved through a projection operation onto a pedagogical constraint subspace:


$$\begin{array}{l} f_{t}^{*}=P_{C}\left({\hat{f}}_{t}\right)=\arg\underset{x\in C}{\min}∥x-{\hat{f}}_{t}{∥}_{2}^{2} \end{array}$$ (23)

where C represents the constraint manifold encoding desired instructional orientations and pedagogical norms, ensuring that the evolving feedback trajectory stays aligned with educational intent while responding to real-time behavioral variations.

#### 3.4.3 Pedagogical reward adaptation

AFOS integrates a pedagogical reward adaptation mechanism designed to reinforce feedback decisions that contribute meaningfully to learner progression. Unlike static reward assignments, this mechanism continuously evaluates the instructional efficacy of feedback by measuring its impact on learner behavior improvement trajectories, adapting the optimization process accordingly. The pedagogical reward function *R*(*b, f*) is formulated to reflect both short-term responsiveness and long-term alignment with instructional objectives. Each feedback *f* is evaluated through a reward function that combines immediate behavioral alignment and trajectory advancement:


$$\begin{array}{l} R\left(b,f\right)=\omega_{1}\cdot \text{Sim}\left(b,\hat{b}\right)+\omega_{2}\cdot \Delta_{\text{engage}}\left(f\right) \end{array}$$ (24)

where $\hat{b}$ is the target behavior, Sim(·) measures semantic alignment, Δ_engage_(*f*) quantifies the increase in engagement post-feedback, and ω_1_, ω_2_ are tunable importance weights.

In medical education, effective feedback refers to guidance that is actionable for the learner within the current training task and that supports the intended clinical reasoning or procedural skill. Effectiveness is not defined only by immediacy, but by whether the feedback is specific to the observed action, linked to the underlying clinical principle, and oriented toward a clear corrective step that can be attempted in the next interaction. Under this view, the term $\text{Sim}\left(b,\hat{b}\right)$ in *R*(*b, f*) represents instructional alignment. It captures whether the feedback content targets the behavior that prompted the intervention and whether it reinforces the correct clinical concept or corrects a misconception reflected in the recognized behavior. The term Δ_engage_(*f*) represents continued participation after feedback, quantified using behavior signals available to the system, such as response timing, attention stability, and interaction frequency under the sensing conditions of the study. This term is used as a proxy for whether feedback delivery maintains learner involvement in the activity, which supports sustained practice during simulation based training.

The weights ω_1_ and ω_2_ are selected to balance instructional alignment and continued engagement under the validation protocol. A larger ω_1_ emphasizes content fidelity and ensures that selected feedback remains coupled to the targeted behavior and the instructional objective, while a nonzero ω_2_ discourages choices that are instructionally correct but reduce subsequent participation. In practice, a small set of candidate weight settings is evaluated on a held out validation split and the final choice is selected based on stable feedback relevance and consistent behavior recognition performance across scenarios. This selection procedure sets reward parameters using measurable system level criteria, while avoiding claims of direct measurement of learning outcomes in the current evaluation.

The system then computes an expected reward over observed behaviors, integrating uncertainty in learner response using a probabilistic expectation:


$$\begin{array}{l} {𝔼}_{b\sim B}\left[R\left(b,f\right)\right]=\overset{n}{\underset{i=1}{\sum }}p\left(b_{i}\right)\cdot R\left(b_{i},f\right) \end{array}$$ (25)

where *p*(*b*_*i*_) is the modeled probability of behavior *b*_*i*_ being observed given prior instructional context. To regularize the feedback signal and ensure pedagogical compliance, AFOS introduces an instructional norm penalty Ω(*f*), which is modeled as a deviation from expert-defined feedback vectors *f*_ref_:


$$\begin{array}{l} \Omega \left(f\right)=∥f-f_{\text{ref}}{∥}^{2} \end{array}$$ (26)

This term penalizes feedback that significantly diverges from established pedagogical guidelines. The final adaptive optimization objective combines reward maximization and norm adherence, solved by selecting the feedback signal f^*^ that achieves optimal instructional impact under regularization:


$$\begin{array}{l} f^{*}=\arg\underset{f}{\max}\left[\overset{n}{\underset{i=1}{\sum }}p\left(b_{i}\right)\cdot R\left(b_{i},f\right)-\lambda \cdot ∥f-f_{\text{ref}}{∥}^{2}\right] \end{array}$$ (27)

This ensures that feedback dynamically evolves in response to learner outcomes while respecting pedagogical structure and instructional intent, making the optimization process both behavior-sensitive and goal-consistent.

## 4 Experimental setup

### 4.1 Dataset

The Medical Teaching Interaction Dataset (39) is a comprehensive collection designed to facilitate the study of interactions within medical teaching environments. This dataset includes a wide range of recorded sessions from various medical training scenarios, capturing both verbal and non-verbal communication between instructors and students. The data is meticulously annotated to highlight key interaction points, such as question-and-answer exchanges, feedback moments, and collaborative problem-solving instances. This level of detail allows researchers to analyze the dynamics of teaching methods and their effectiveness in real-time educational settings. The dataset is particularly valuable for developing machine learning models aimed at improving automated feedback systems and enhancing the overall quality of medical education.

The Attention-Based Behavior Recognition Dataset (40) focuses on capturing and analyzing human attention patterns in various behavioral contexts. This dataset comprises video recordings and sensor data from controlled experiments where participants engage in tasks requiring varying levels of attention and cognitive load. Each session is annotated with attention markers, indicating shifts in focus and engagement levels. The dataset serves as a critical resource for developing algorithms that can accurately recognize and predict attention-based behaviors, which are essential for applications in human-computer interaction, surveillance, and cognitive research. By providing a rich set of features related to attention dynamics, this dataset supports the advancement of behavior recognition technologies.

The Feedback Optimization in Medical Education Dataset (41) is specifically curated to explore the impact of feedback mechanisms in medical training. It includes a diverse array of feedback scenarios, ranging from peer-to-peer reviews to instructor-led evaluations. The dataset captures both the content and delivery of feedback, along with subsequent changes in learner performance and engagement. This comprehensive collection enables researchers to investigate the optimal conditions for feedback delivery and its effects on learning outcomes. By analyzing this dataset, educational technologists can develop more effective feedback systems that enhance learning efficiency and retention in medical education.

The Interactive Learning Behavior Dataset (42) is designed to study the intricacies of interactive learning environments. It encompasses a variety of educational settings, including traditional classrooms, online platforms, and hybrid models. The dataset includes detailed recordings of learner interactions, both with instructors and peers, as well as with digital learning tools. Annotations highlight key interactive behaviors, such as collaboration, inquiry, and problem-solving. This dataset is instrumental in understanding how interactive elements influence learning processes and outcomes. Researchers can leverage this data to design more engaging and effective educational technologies that cater to diverse learning needs and preferences.

To improve transparency and support reproducibility, we provide a summary of all datasets used in this study in Table 1. This includes key details such as the number of participants, number of recorded sessions, data modalities, and annotation types. The Medical Teaching Interaction Dataset captures audio-visual and textual exchanges in structured instructional settings, annotated with engagement states and feedback events. The Attention-Based Behavior Recognition Dataset focuses on fine-grained attention dynamics, combining video, EEG signals, and motion sensor data to capture subtle behavioral shifts. The Feedback Optimization in Medical Education Dataset offers annotated feedback scenarios that allow for evaluation of adaptive feedback strategies across diverse learner profiles. The Interactive Learning Behavior Dataset includes multi-role learning tasks annotated for collaboration and inquiry behaviors. These datasets represent a variety of medical and educational interaction contexts, ensuring the robustness and generalizability of the proposed framework under different pedagogical conditions.

**Table 1:** Summary of datasets used in the experiments.

**Dataset**	**Participants**	**Sessions**	**Modalities**	**Annotations**
Medical Teaching Interaction Dataset	58	420	Video, audio, text	Engagement states, feedback events
Attention-Based Behavior Recognition Dataset	35	160	Video, EEG, motion sensors	Attention shifts, behavioral tags
Feedback Optimization in Medical Education Dataset	42	300	Text, audio, feedback logs	Feedback type, learner response
Interactive Learning Behavior Dataset	75	500+	Video, mouse	Collaboration, problem-solving labels

Among the datasets summarized in Table 1, engagement and comprehension labels are explicitly included in the Medical Teaching Interaction Dataset and the Interactive Learning Behavior Dataset. In the former, learner engagement is annotated based on facial expressions, gesture analysis, and speaking patterns, while comprehension states are inferred from question, response accuracy and temporal delays. These labels are critical for modeling both real-time learner states and longitudinal understanding levels, and they directly inform the adaptive feedback selection process. The Attention-Based Behavior Recognition Dataset includes implicit indicators related to attention and cognitive load, which are aligned with engagement estimation but do not include discrete comprehension labels. The Feedback Optimization Dataset focuses on feedback-response dynamics and does not contain direct engagement or comprehension tags. In our framework, engagement and comprehension representations are primarily learned from the two aforementioned datasets that include these structured labels.

### 4.2 Experimental details

All experiments were conducted on an NVIDIA Tesla V100 GPU (32 GB memory) with CUDA 11.1 to support stable large-scale training. The backbone network was initialized with ImageNet-pretrained weights to improve optimization stability and accelerate convergence. Unless otherwise stated, a batch size of 64 was used throughout all experiments, as it provided the most reliable training behavior in preliminary trials under the available GPU memory budget. The initial learning rate was set to 1 × 10^−3^ and decayed by a factor of 10 every 10 epochs. To further stabilize early-stage optimization, a warm-up strategy was applied during the first five epochs, gradually increasing the learning rate from a small value to the target setting. An adaptive gradient-based optimizer was used for all training runs.

To enhance input diversity and reduce the risk of overfitting, standard data augmentation techniques were employed, including random horizontal flipping, random cropping, and controlled color perturbation. Model performance was evaluated using four complementary metrics: F1 score, AUC, Accuracy, and Recall, providing a multi-perspective assessment of classification reliability.

Hyperparameter selection followed a two-stage protocol. We first performed coarse range testing on a subset of the Medical Teaching Interaction Dataset to identify stable configurations, followed by fine-tuning on a held-out validation set. Learning rates were evaluated in the range {1 × 10^−4^, 5 × 10^−4^, 1 × 10^−3^, 5 × 10^−3^}, with 1 × 10^−3^ yielding the best trade-off between convergence speed and loss stability. Batch sizes of {32, 64, 128} were compared, and 64 was selected due to its stable training behavior without causing memory overflow. For the multi-head self-attention module, the number of attention heads was chosen from {4, 8, 12}, and 8 heads achieved the best validation performance without significant computational overhead. Embedding dimensionality was tested in {128, 256, 512}, with 256 selected to balance representational capacity and overfitting risk. Dropout rates were tuned between 0.1 and 0.5, and a value of 0.3 provided the most consistent generalization. For robustness, all experiments were repeated five times, and hyperparameter selection was based on the average F1 score and AUC on the validation set. Test set information was not used during tuning. To support reproducibility, we release the source code and pretrained model weights, and fix the random seed to reduce variance introduced by stochastic optimization.

### 4.3 Comparison with SOTA methods

The experimental outcomes presented in Tables 2 and 3 reveal a clear performance advantage achieved by the proposed method, and these gains align well with the core research theme of enhancing spatiotemporal understanding in complex behavioral scenarios. In Table 2, the results on the Medical Teaching Interaction Dataset show that the proposed system delivers higher accuracy, precision, recall, and AUC than competing models, demonstrating its ability to capture subtle teaching gestures and instructional patterns. These improvements arise from the integration of multi scale attention, which enriches spatiotemporal representations by emphasizing informative regions while maintaining awareness of global structural context. This design avoids the limitations of purely convolutional models that tend to overlook wide range dependencies. The smoother optimization behavior observed in the training process can be attributed to a gradual adjustment schedule that stabilizes parameter updates and reduces oscillations during learning. Evidence of enhanced robustness becomes even clearer on the Attention Based Behavior Recognition Dataset, where markedly stronger recognition stability indicates improved generalization across different instructional settings. The specialized feature extraction pipeline contributes to this outcome by balancing detailed local motion cues with broader contextual patterns. The observed superiority in metrics such as recall and AUC suggests that the proposed loss formulation mitigates class imbalance more effectively than conventional techniques, enabling the model to detect rare but critical behavioral events with greater consistency. From a modular perspective, removing the attention enriched representation extractor significantly weakens recognition accuracy, while eliminating the temporal refinement component reduces stability across sequential frames, which confirms the value of each architectural element.

**Table 2:** Evaluation of models on Medical Teaching Interaction Dataset and Attention-Based Behavior Recognition Dataset.

**Model**	**Medical Teaching Interaction Dataset**				**Attention-Based Behavior Recognition Dataset**			
	**Accuracy**	**Precision**	**Recall**	**AUC**	**Accuracy**	**Precision**	**Recall**	**AUC**
ResNet (43)	85.67 ± 0.52	84.92 ± 0.63	85.14 ± 0.58	84.75 ± 0.47	87.23 ± 0.49	86.45 ± 0.60	86.78 ± 0.55	86.12 ± 0.50
ViT (44)	86.45 ± 0.47	85.78 ± 0.54	85.96 ± 0.61	85.32 ± 0.44	88.34 ± 0.42	87.56 ± 0.57	87.89 ± 0.52	87.23 ± 0.48
I3D (45)	84.92 ± 0.55	84.15 ± 0.60	84.37 ± 0.57	83.89 ± 0.53	86.78 ± 0.51	86.02 ± 0.64	86.25 ± 0.59	85.67 ± 0.54
BLIP (46)	87.12 ± 0.48	86.34 ± 0.59	86.56 ± 0.54	86.02 ± 0.46	88.92 ± 0.45	88.15 ± 0.58	88.37 ± 0.53	87.71 ± 0.49
DenseNet (47)	85.89 ± 0.50	85.12 ± 0.62	85.34 ± 0.59	84.78 ± 0.51	87.56 ± 0.47	86.78 ± 0.61	87.01 ± 0.56	86.45 ± 0.52
MobileNet (48)	86.78 ± 0.46	86.01 ± 0.57	86.23 ± 0.52	85.89 ± 0.49	88.45 ± 0.44	87.67 ± 0.55	87.89 ± 0.50	87.34 ± 0.46
Ours	**89.34** **±** 0.40^*^	**88.56** **±** 0.49^*^	**88.78** **±** 0.45^*^	**88.23** **±** 0.43^**^	**91.02** **±** 0.38^**^	**90.34** **±** 0.48^**^	**90.56** **±** 0.44^**^	**90.01** **±** 0.41^**^

Results are reported as mean ± standard deviation, with 95% confidence intervals estimated via bootstrap (1,000 iterations).

Statistical significance relative to our method is assessed using a paired two-tailed *t*-test across five runs.

^*^ indicates *p* < 0.05, ^**^ indicates *p* < 0.01.

The bolded values represent the optimal values.

**Table 3:** Evaluation of models on Feedback Optimization in Medical Education Dataset and Interactive Learning Behavior Dataset.

**Model**	**Feedback Optimization in Medical Education Dataset**				**Interactive Learning Behavior Dataset**			
	**Accuracy**	**Recall**	**F1 score**	**AUC**	**Accuracy**	**Recall**	**F1 score**	**AUC**
ResNet (43)	84.56 ± 0.58	83.92 ± 0.67	83.15 ± 0.72	83.48 ± 0.65	87.34 ± 0.62	86.78 ± 0.70	86.01 ± 0.68	86.29 ± 0.63
ViT (44)	85.89 ± 0.47	85.23 ± 0.55	84.46 ± 0.60	84.79 ± 0.52	88.67 ± 0.51	88.12 ± 0.59	87.35 ± 0.57	87.63 ± 0.54
I3D (45)	86.12 ± 0.49	85.47 ± 0.58	84.70 ± 0.63	85.03 ± 0.56	88.90 ± 0.53	88.35 ± 0.61	87.58 ± 0.60	87.86 ± 0.57
BLIP (46)	85.45 ± 0.52	84.81 ± 0.61	84.04 ± 0.66	84.37 ± 0.59	88.23 ± 0.56	87.68 ± 0.64	86.91 ± 0.62	87.19 ± 0.59
DenseNet (47)	86.78 ± 0.46	86.13 ± 0.54	85.36 ± 0.59	85.69 ± 0.51	89.56 ± 0.50	89.01 ± 0.58	88.24 ± 0.56	88.52 ± 0.53
MobileNet (48)	85.67 ± 0.50	85.02 ± 0.59	84.25 ± 0.64	84.58 ± 0.57	88.45 ± 0.54	87.90 ± 0.62	87.13 ± 0.60	87.41 ± 0.57
Ours	**89.12** **±** 0.44^*^	**88.47** **±** 0.53^**^	**87.70** **±** 0.58^**^	**88.03** **±** 0.55^**^	**91.34** **±** 0.48^**^	**90.79** **±** 0.56^**^	**90.02** **±** 0.54^**^	**90.30** **±** 0.51^**^

Each metric is shown as mean ± standard deviation, along with 95% confidence intervals.

Significance is tested using a paired t-test across five trials.

^*^ denotes statistically significant difference at *p* < 0.05, ^**^ at *p* < 0.01, compared with our method.

The bolded values represent the optimal values.

The findings in Table 3 further validate these conclusions across the Feedback Optimization in Medical Education Dataset and the Interactive Learning Behavior Dataset, both of which exhibit more diverse interaction styles and richer temporal variation. The proposed method again surpasses all baselines, showing strong improvements in accuracy, recall, F1 score, and AUC. These gains are supported by targeted data augmentation, such as controlled color shifts and spatial perturbations, which expand data diversity and reduce overfitting. The hybrid gradient update strategy also contributes by guiding the optimization trajectory more smoothly and supporting steadier convergence. On the Interactive Learning Behavior Dataset, the model benefits from the pose oriented refinement mechanism, which improves the precision of motion interpretation and supports more accurate estimation of subtle engagement patterns. When combined with the attention driven extractor, this refinement enables more reliable modeling of student and instructor interaction dynamics. Ablation analysis indicates that removing either the multi scale attention or the temporal consistency component leads to clear degradation in F1 score and AUC, confirming that both modules play essential roles in strengthening spatiotemporal reasoning. These results indicate that the proposed approach improves behavior recognition performance across distinct interaction conditions, while the next section discusses how such improvements can be interpreted in educational terms.

### 4.4 Ablation study

The ablation analysis highlights how each major element of the overall system contributes to its effectiveness, with particular attention given to the Attention-Enhanced Spatiotemporal Embedding, the Context-Aware Attention Calibration, and the Pedagogical Reward Adaptation. Tables 4 and 5 summarize the behavior of the system once individual elements are modified or removed. The configuration containing every module consistently yields the strongest results, illustrating how these parts reinforce one another. Excluding the Attention-Enhanced Spatiotemporal Embedding leads to a marked reduction in predictive reliability, emphasizing its significance in transforming raw features into richer representations capable of reflecting the subtleties present in medical instructional interactions.

**Table 4:** Evaluation of model variants on Medical Teaching Interaction Dataset and Attention-Based Behavior Recognition Dataset.

**Model**	**Medical Teaching Interaction Dataset**				**Attention-Based Behavior Recognition Dataset**			
	**Accuracy**	**Precision**	**Recall**	**AUC**	**Accuracy**	**Precision**	**Recall**	**AUC**
w./o. Attention-Enhanced Spatiotemporal Embedding	87.45 ± 0.48	86.78 ± 0.57	87.01 ± 0.53	86.56 ± 0.50	89.12 ± 0.46	88.34 ± 0.55	88.67 ± 0.51	88.12 ± 0.47
w./o. Context-Aware Attention Calibration	88.12 ± 0.45	87.34 ± 0.54	87.56 ± 0.50	87.23 ± 0.47	89.78 ± 0.43	89.01 ± 0.52	89.23 ± 0.48	88.67 ± 0.44
w./o. Pedagogical Reward Adaptation	88.67 ± 0.43	87.89 ± 0.52	88.12 ± 0.49	87.78 ± 0.45	90.23 ± 0.41	89.45 ± 0.50	89.67 ± 0.46	89.12 ± 0.42
Ours	**89.34** **±** **0.40**	**88.56** **±** **0.49**	**88.78** **±** **0.45**	**88.23** **±** **0.43**	**91.02** **±** **0.38**	**90.34** **±** **0.48**	**90.56** **±** **0.44**	**90.01** **±** **0.41**

The bolded values represent the optimal values.

**Table 5:** Evaluation of model variants on Feedback Optimization in Medical Education Dataset and Interactive Learning Behavior Dataset.

**Model**	**Feedback Optimization in Medical Education Dataset**				**Interactive Learning Behavior Dataset**			
	**Accuracy**	**Recall**	**F1 Score**	**AUC**	**Accuracy**	**Recall**	**F1 Score**	**AUC**
w./o. Attention-Enhanced Spatiotemporal Embedding	87.45 ± 0.52	86.89 ± 0.61	86.12 ± 0.66	86.45 ± 0.59	90.23 ± 0.56	89.68 ± 0.64	88.91 ± 0.62	89.19 ± 0.59
w./o. Context-Aware Attention Calibration	88.12 ± 0.49	87.57 ± 0.58	86.80 ± 0.63	87.13 ± 0.56	90.90 ± 0.53	90.35 ± 0.61	89.58 ± 0.60	89.86 ± 0.57
w./o. Pedagogical Reward Adaptation	87.78 ± 0.50	87.23 ± 0.59	86.46 ± 0.64	86.79 ± 0.57	90.56 ± 0.54	90.01 ± 0.62	89.24 ± 0.60	89.52 ± 0.57
Ours	**89.12** **±** **0.44**	**88.47** **±** **0.53**	**87.70** **±** **0.58**	**88.03** **±** **0.55**	**91.34** **±** **0.48**	**90.79** **±** **0.56**	**90.02** **±** **0.54**	**90.30** **±** **0.51**

The bolded values represent the optimal values.

A similar trend emerges for the Context-Aware Attention Calibration, which plays a central role in constructing context-aware representations through attention-driven weighting. When this layer is omitted, performance declines noticeably, demonstrating its importance for spotlighting informative signals and filtering out irrelevant or noisy cues. This selective focus mechanism allows the model to maintain stability even in challenging or cluttered environments.

The Pedagogical Reward Adaptation contributes an additional dimension by adjusting instructional responses according to ongoing behavioral patterns. Removing this component reduces consistency and weakens generalization, as indicated in Table 5. Its refinement process supports feedback adjustments that remain aligned with learners' immediate states and instructional objectives. The findings also show that each module contributes a distinct function within the overall system. In combination, the modules form a cohesive framework whose measured performance exceeds that of any single component, which supports the value of the integrated design.

The proposed framework contains multiple components, yet this design choice is motivated by the characteristics of multimodal educational interactions and the requirements of behavior informed feedback. A simpler architecture can model a subset of the problem, but it often struggles to address selective evidence use across modalities, temporal context accumulation, and the coupling between recognition and feedback decisions. Convolutional models are effective for local spatial pattern extraction, yet they are limited in representing longer interaction dependencies and may treat all input regions with similar priority. Recurrent sequence models can encode temporal order, yet they may not provide explicit mechanisms to emphasize salient moments, integrate longer behavioral history in a controlled manner, or support calibration under varying instructional contexts. The attention and memory related modules are included to highlight informative cues, preserve context across interaction sequences, and maintain consistency between detected behaviors and the feedback selected by the system. This motivation is consistent with the ablation results reported in Tables 4, 5, where removing key modules leads to clear degradation in measured performance, indicating that each component contributes to the overall capability.

Computational cost is also an important practical consideration. The full model requires more computation than a simplified baseline due to multimodal processing and temporal reasoning. This work evaluates the framework under controlled experimental conditions, while deployment oriented settings may require careful choices of input resolution, sequence length, and model capacity to meet latency and resource constraints. The modular structure provides flexibility for practical use, since components can be configured to match the available sensing modalities and computational budget. Efficiency oriented variants, such as reduced capacity attention, shorter temporal windows, or compressed parameterization, can be explored when the target setting demands lower overhead, while preserving the core behavior recognition and feedback mapping functions.

### 4.5 Educational interpretation of the results

In medical education, the terms learner engagement, instructional behavior, and feedback effectiveness refer to pedagogical constructs that are related to, but not identical to, technical prediction accuracy. Learner engagement refers to observable and inferred indicators of a learner's attentional focus and cognitive investment during learning activities. In the present setting, engagement can be reflected through behaviors such as sustained visual attention to task relevant content, timely responses to prompts, and active participation in discussions or practice steps. Instructional behavior refers to the actions and strategies used by instructors to guide learning, including pacing decisions, prompting and questioning, repetition and clarification, and responses to learner cues that signal confusion or low participation. Feedback effectiveness refers to how well the feedback delivered by a system matches the learner's immediate needs and the instructional intent, supports conceptual clarity, and helps address misunderstandings or skill gaps in a timely manner.

The reported metrics in this study, including accuracy, F1 score, and AUC, evaluate the system's ability to recognize behavior patterns and to support feedback selection under the defined experimental protocol. Their educational relevance arises from the role that recognition quality plays in downstream instructional support. A higher F one score in behavior recognition indicates more reliable detection of behavior categories that are used as triggers for guidance, reducing missed cases of disengagement and reducing false alarms that could distract instruction. Improvements in AUC indicate stronger discrimination across varied interaction conditions, which supports more consistent performance when teaching scenarios differ in difficulty, modality, or interaction style. When the system produces more stable recognition outputs, the feedback module can base its recommendations on more trustworthy behavioral evidence, which improves the likelihood that suggested interventions are aligned with the observed learning state and the current teaching context.

From a practical perspective, these technical gains can help instructors and learners in simulated or hybrid medical teaching settings by enabling more timely identification of moments that may require clarification, reinforcement, or practice adjustments. The framework can support rapid remediation of missed steps, reinforcement of weak areas identified through interaction patterns, and adaptive sequencing of practice activities according to observed behavior trajectories. The current evaluation does not directly measure learning outcomes such as exam performance, long term retention, or clinical competence, yet it strengthens system level recognition and feedback processes that are commonly used as prerequisites for effective instructional support and that can be examined in future educational studies.

## 5 Conclusions and future work

The integration of the Attention Driven Interactive Behavior Recognition Model and the Adaptive Feedback Optimization Strategy offers a coherent approach for intelligent medical teaching systems. By incorporating attention mechanisms into multimodal behavior analysis, the framework improves the capture of instructional interactions and highlights salient temporal and contextual cues. These properties support more reliable identification of learning related behaviors and improve the precision of behavior oriented pedagogical analysis. The feedback optimization strategy maps recognized behavioral patterns to adaptive instructional responses that remain consistent with observed interaction states and predefined educational objectives. Quantitative evaluations on multiple datasets show consistent improvements on measured behavior recognition metrics and feedback relevance when compared with existing methods. These results suggest that attention driven multimodal modeling can strengthen system level recognition and feedback generation, while direct evidence of improved learning outcomes or instructional effectiveness requires dedicated educational studies that are not included in the current evaluation.

Future developments can refine and extend the capabilities of the Attention Driven Interactive Behavior Recognition Model and the Adaptive Feedback Optimization Strategy. Attention mechanisms provide measurable gains in the current experiments, yet computational cost remains an important consideration, particularly when serving large cohorts or operating in highly dynamic learning settings. Future research can examine model compression, lightweight attention designs, and more efficient multimodal fusion to improve practicality for real time use. The framework can also be extended to represent learner specific factors, such as individualized cognitive profiles and longitudinal learning analytics, to support more adaptive guidance across sessions. Broader evaluation in varied instructional contexts and deployment oriented studies can help clarify robustness, usability, and educational impact beyond system level metrics. Integration with immersive simulation platforms, virtual reality systems, and reinforcement learning based optimization may further improve adaptability and responsiveness of medical teaching systems.

While the proposed framework shows promising performance under controlled experimental conditions, several limitations should be considered for practical application. The current design assumes relatively stable and high quality multimodal input signals, which may not always be available in real classroom settings. Noise from sensors, partial observation of learner behavior, and missing interaction cues may affect robustness and consistency. The evaluation in this study focuses on behavior recognition accuracy and feedback relevance, while direct measurement of learning outcomes and knowledge retention is not included. Assessing educational impact would require longitudinal studies with diverse learner groups, which are beyond the present scope. Scalability also remains an important consideration. Continuous multimodal attention processing and feedback optimization can introduce computational overhead that may challenge real time deployment in large teaching environments or settings with limited computing resources. Addressing these issues will be important for extending the applicability of the framework.

## Data Availability

The original contributions presented in the study are included in the article/supplementary material, further inquiries can be directed to the corresponding author.
